# Reduction of Claustrophobia with Short-Bore versus Open Magnetic Resonance Imaging: A Randomized Controlled Trial

**DOI:** 10.1371/journal.pone.0023494

**Published:** 2011-08-22

**Authors:** Judith Enders, Elke Zimmermann, Matthias Rief, Peter Martus, Randolf Klingebiel, Patrick Asbach, Christian Klessen, Gerd Diederichs, Moritz Wagner, Ulf Teichgräber, Thomas Bengner, Bernd Hamm, Marc Dewey

**Affiliations:** 1 Department of Radiology, Charité, Medical School, Humboldt Universität zu Berlin and Freie Universität Berlin, Berlin, Germany; 2 Department of Biostatistics and Clinical Epidemiology, Charité, Medical School, Humboldt Universität zu Berlin and Freie Universität Berlin, Berlin, Germany; 3 Division of Neuroradiology, Department of Radiology, Charité, Medical School, Humboldt-Universität zu Berlin and Freie Universität Berlin, Berlin, Germany; 4 Department of Clinical Psychology, Charité, Medical School, Humboldt-Universität zu Berlin and Freie Universität Berlin, Berlin, Germany; Federal University of Rio de Janeiro, Brazil

## Abstract

**Background:**

Claustrophobia is a common problem precluding MR imaging. The purpose of the present study was to assess whether a short-bore or an open magnetic resonance (MR) scanner is superior in alleviating claustrophobia.

**Methods:**

Institutional review board approval and patient informed consent were obtained to compare short-bore versus open MR. From June 2008 to August 2009, 174 patients (139 women; mean age = 53.1 [SD 12.8]) with an overall mean score of 2.4 (SD 0.7, range 0 to 4) on the Claustrophobia Questionnaire (CLQ) and a clinical indication for imaging, were randomly assigned to receive evaluation by open or by short-bore MR. The primary outcomes were incomplete MR examinations due to a claustrophobic event. Follow-up was conducted 7 months after MR imaging. The primary analysis was performed according to the intention-to-treat strategy.

**Results:**

With 33 claustrophobic events in the short-bore group (39% [95% confidence interval [CI] 28% to 50%) versus 23 in the open scanner group (26% [95% CI 18% to 37%]; *P* = 0.08) the difference was not significant. Patients with an event were in the examination room for 3.8 min (SD 4.4) in the short-bore and for 8.5 min (SD 7) in the open group (*P* = 0.004). This was due to an earlier occurrence of events in the short-bore group. The CLQ suffocation subscale was significantly associated with the occurrence of claustrophobic events (*P* = 0.003). New findings that explained symptoms were found in 69% of MR examinations and led to changes in medical treatment in 47% and surgery in 10% of patients. After 7 months, perceived claustrophobia increased in 32% of patients with events versus in only 11% of patients without events (*P = *0.004).

**Conclusions:**

Even recent MR cannot prevent claustrophobia suggesting that further developments to create a more patient-centered MR scanner environment are needed.

**Trial Registration:**

ClinicalTrials.gov NCT00715806

## Introduction

Magnetic resonance (MR) imaging has been rated by leading general internists to be, together with computed tomography (CT), the most important medical innovation of the last 25 years [1]. However, MR imaging can be severely hampered by claustrophobia induced by confinement in the long narrow bore of conventional scanners and further unpleasant aspects of the examination such as scanner noise and vibration [2], [3], [4], [5]. Anxious patients suffer from claustrophobia during MR imaging in up to 35% of all cases [2], [6], [7], and claustrophobic events can lead to abortion of imaging or require sedation for its completion. This situation decreases diagnostic yield, limits patient acceptance, and reduces workflow. Moreover, conscious sedation to alleviate claustrophobia involves significant risks [8], [9], [10].

Thus, claustrophobia is a common challenge for performing MR imaging and has been investigated in several large non-randomized studies [11], [12], [13]. It was found that between 1 and 15% of all MR examinations in unselected patients on conventional scanners cannot be completed because of claustrophobia or require conscious sedation to be completed [12]. Cognitive behavioral treatment, as by exposure to claustrophobic stimuli, is one effective approach to face the problem [14], [15]. Structured empathic attention by trained staff and instructing patients to self-hypnotic relaxation have also shown to reduce anxiety during MR imaging and other medical procedures [16], [17], [18]. However, such options may not usually be available. Another approach to lower the rate of claustrophobic events is thus to improve the design of MR scanners. Two recent concepts are a more open panoramic scanner and a short-bore configuration [11], [12], [19], [20]. We compared these two scanner configurations in a randomized controlled trial in patients with an increased risk for claustrophobic events in MR imaging.

## Methods

The supporting CONSORT checklist is available as supporting information; see Checklist S1. The detailed trial protocol has been published [21].

### Ethics statement

Approval was obtained from the institutional review board at Charité, Berlin. After complete description of the study, written informed consent was obtained from all participants prior to randomization.

### Study design

**Figure 1:**
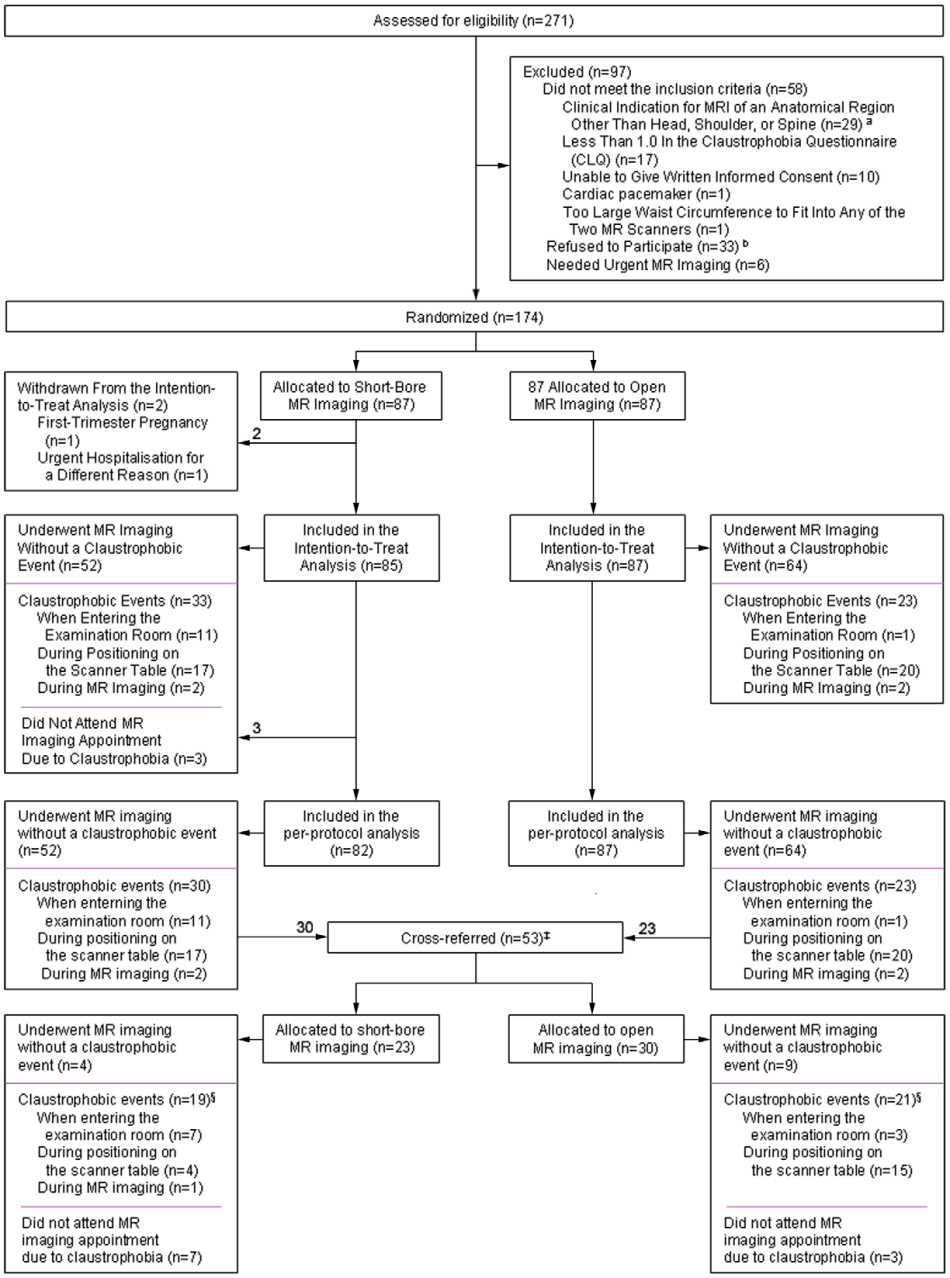
Screening, randomization, and claustrophobic events in the study. ^*^ Of the 29 patients who had a clinical indication for MR imaging of an anatomical region other than head, shoulder, or spine, 10 needed MR imaging of the knee, 6 of the hip, 3 of the petrous bone, 2 of the iliosacral joints, 2 of the breasts, one each of the whole body, the lower leg, the sternum, the neck, the spleen, and the eye. ^†^ Of the 33 patients who were eligible but declined to participate, 14 considered study participation too time-consuming, 6 had an appointment for open MR imaging elsewhere, 6 decided not to undergo MR imaging despite clinical indications, and 7 gave no reason. ^‡^ Patients were cross-referred for a second MR examination on the other scanner if they could not bear imaging on the first scanner in order to avoid the risks of conscious sedation. Patients were offered cross-referral within one week. If patients could not bear MR imaging on the second scanner either, conscious sedation was performed according to the guideline of the American Society of Anesthesiology to reduce risks [25]. ^§^ Of the 30 patients who did attend the MR appointment and had claustrophobic events before or during the second MR imaging, 26 underwent MR imaging with conscious sedation using a mean of 2.6 mg (SD = 1) midazolam IV (sedation success rate 100%, no adverse events). Four patients rejected conscious sedation and could not undergo MR imaging.

Between June 19, 2008 and August 14, 2009, we performed a prospective single-center parallel-group randomized controlled trial in 174 patients in a university hospital (**Figure 1**). This trial was conducted and is reported in accordance with the CONSORT guidelines for non-pharmacological trials [22].

### Participants

Patients referred to our hospital for MR imaging were screened for eligibility by author JE, EZ or MR if they reported or feared to feel claustrophobic in MR scanners (or if the referring physician provided this information). Enrolled patients were required to have a clinical indication for MR imaging of the head, spine, or shoulder as well as a total mean score of at least 1.0 in the Claustrophobia Questionnaire (CLQ) (**Table 1**) [23]. The CLQ consists of 26 items which assess two separate but related fears hypothesized to comprise claustrophobia: the fear of suffocation and the fear of restriction. 14 items of the CLQ are assigned to the suffocation and 12 items to the restriction scale. For each of the 26 items of the CLQ, anxiety is rated on a scale from 0 (not at all anxious) to 4 (extremely anxious). Thus, the total mean CLQ score can range from 0 to 4. Exclusion criteria were absolute or relative contraindications to MR imaging [24], body weight of more than 200 kg (due to safety restrictions of the MR tables), and age below 18 years.

**Table 1:** Anxiety measures and MR imaging characteristics of the 174 patients enrolled in the study.

	Short-bore MR group (n = 87)	Open MR group (n = 87)	*P* Value
**Claustrophobia and Anxiety**			
Claustrophobia Questionnaire (CLQ) [23]			
Mean overall value	2.4 (SD 0.8)	2.4 (SD 0.7)	0.73
Restriction subscale	2.9 (SD 0.8)	2.9 (SD 0.7)	0.9
Suffocation subscale	2 (SD 0.9)	1.9 (SD 0.8)	0.66
Claustrophobia VAS*	66 (SD 23.2)	63.9 (SD 25.3)	0.6
Claustrophobia preventing prior scheduled MR imaging	33 (38)	33 (38)	1
Abortion of prior MR imaging due to claustrophobia	8 (5)	11 (6)	0.47
Sedation for prior MR imaging required	7 (4)	6 (3)	0.77
Belief in successful completion of MR imaging			0.71
Yes	34 (39)	31 (36)	
No	8 (9)	6 (7)	
Undecided	45 (52)	50 (58)	
Anxious about the possible diagnostic findings of MR imaging			0.72
Yes	18 (21)	21 (24)	
No	57 (66)	57 (66)	
Undecided	12 (14)	9 (10)	
**MR imaging**			
Prior MR imaging	69 (79)	74 (85)	0.32
Region of MR imaging†			0.92
Head	19 (22)	22 (25)	
Shoulder	15 (17)	12 (14)	
Cervical spine	16 (18)	19 (22)	
Lumbar spine	32 (37)	29 (33)	
Whole spine	5 (6)	5 (6)	
Appropriateness‡			0.77
Inappropriate	0 (0)	0 (0)	
Equivocal	17 (20)	16 (18)	
Appropriate	62 (71)	60 (69)	
Necessary	8 (9)	11 (13)	
Pain§	18.8 (SD 26)	32.2 (SD 34.7)	0.01
Noise§	59.7 (SD 21.9)	67.3 (SD 22.9)	0.63
Anxiety§	61.9 (SD 33.7)	58.3 (SD 31.6)	0.51

Values are arithmetic mean (standard deviation [SD]) or number (%). Percentages may not total 100% because of rounding.

*Patients were asked to report their perceived level of claustrophobic anxiety at initial assessment using a horizontal and non-marked (0–100 mm) visual analogue scale (VAS).

† For MR imaging of the cervical spine, the shoulder, and the head, patients were examined head-first because feet-first imaging, which alleviates claustrophobia by a factor of more than 10, was possible only for lumbar spine imaging in both groups [12].

‡ Appropriateness of the indication for MR imaging was assessed according to the Appropriateness Criteria of the American College of Radiology [33], [34], [35], [36], [37].

§ The pain, noise, and anxiety levels patients experienced during MR imaging were assessed directly after the scan using horizontal and non-marked (0–100 mm) visual analogue scales.

### Randomization and interventions

**Figure 2:**
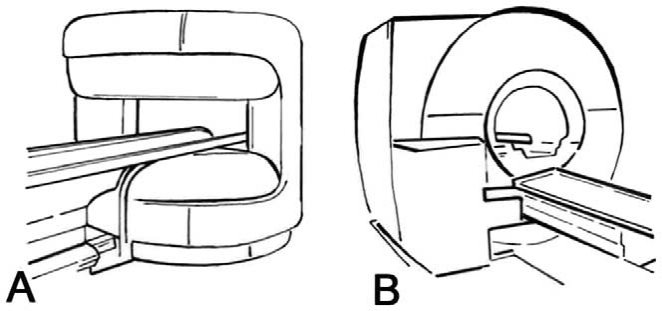
Design of the open and short-bore MR scanners to which patients were randomized in the study. **A.** Open panoramic MR system [19]. **B.** Short-bore MR system. [12]. Figure from [21].

Eligible patients were randomly assigned (computer-generated sequence) in a 1∶1 ratio to: 1) MR imaging in an open panoramic state-of-the-art scanner with a vertical magnetic field, 1-T field strength, up to 26 mT/m gradient strength, maximum acoustic noise of 150 dB(A), and a 0.45 m high and 1.6 m wide patient aperture (0.7 m wide patient table) (Panorama, Philips Medical Systems) [19], or 2) MR imaging in a short-bore state-of-the-art scanner with 1.5-T field strength, up to 45 mT/m gradient strength, 97% noise reduction to below 99 dB(A), and a conical wide (0.6 m) and short (1.5 m) bore (Magnetom Avanto, Siemens Medical Solutions, **Figure 2**) [12]. Randomization was not stratified and allocation was concealed (using sealed envelopes). Patients could not be blinded to the assigned study group due to the MR imaging setting. If patients did not complete imaging in their assigned MR scanner due to claustrophobia, this was counted as an event. These patients were offered imaging in the other scanner within one week in order to avoid the risks of conscious sedation (cross-referral, **Figure 1**). Only patients who could not undergo MR imaging in either of the two scanners received conscious sedation (using midazolam IV) during the second scan according to the American Society of Anaesthesiology guideline [25].

### Assessment instruments

Claustrophobia, general anxiety, symptoms of depression, and health-related quality of life were assessed by having all patients complete the following validated written self-report questionnaires immediately after enrolment: the Agoraphobic Cognitions Questionnaire (ACQ) [26], the Body Sensations Questionnaire (BSQ) [26], the Mobility Inventory (MI) [26], the Spielberger State-Trait Anxiety Inventory (STAI) [27], the Fear Survey Schedule (FSS-III) [28], the Beck Depression Inventory (BDI-II) [29], the EuroQol (EQ-5D) [30], and the Claustrophobia Questionnaire (CLQ) [23]. Patients were asked to report their perceived level of claustrophobia at initial assessment using a horizontal and non-marked (0–100 mm) visual analogue scale [21]. The pain, anxiety, and noise levels patients experienced during imaging were assessed directly after the scan using horizontal non-marked (0–100 mm) visual analogue scales (**Table 1**).

### Follow-up

Seven months after MR imaging, patients were asked to again fill in the self-report questionnaires which were used for initial assessment and to report their current perceived level of claustrophobia using a visual analogue scale. A custom-made questionnaire addressing patients' clinical outcome was administered in order to assess the clinical relevance of the MR findings for subsequent management.

### Outcome measures

An event for the primary hypothesis was defined as the inability of a patient to complete an examination on the assigned MR scanner due to claustrophobia. Events were assessed by two research staff members who had to be present during MR imaging and thus could not be blinded to the study group. Secondary objectives were to analyze the duration of MR imaging, the time at which events occur during the MR examination, the predictive value of validated questionnaires and patient characteristics for claustrophobic events [23], [26], [27], [28], [29], [30], and follow-up results.

### Statistical analysis

Since open MR imaging has been shown in pilot studies to have a high potential to reduce claustrophobia [19], [20], our trial was designed to investigate whether an open panoramic MR scanner is superior to a short-bore MR scanner in reducing the occurrence of claustrophobic events with a statistical power of 80% and an α-level of 0.05. We expected claustrophobia rates in this high-risk patient cohort of 20% in the control group and 5% in the intervention group (15% absolute risk reduction), based on event rates in recent non-randomized studies [12], [19], [20]. Thus, with 82 evaluable patients per group the desired power of 80% was achieved. Conservatively taking into account an expected drop-out rate of 5%, a total of 174 patients, 87 per group, had to be allocated.

The primary analysis was performed according to the intention-to-treat strategy. The chi-squared, the Mann-Whitney rank sum, and the paired and unpaired t-test were used as appropriate for categorical and continuous variables. Differences between rates were examined with chi-squared tests. Confidence intervals (CIs) for absolute and relative risk reduction were calculated with the score method [31], [32]. All tests were two-sided and the level of significance was set at 5% (*P* = 0.05). Statistical analyses were conducted using SPSS version 16.0 (Chicago, IL, US).

## Results

A total of 271 patients were screened, 213 were eligible, and 174 were finally enrolled, 87 in the short-bore MR group and 87 in the open MR group (**Figure 1**).

### Baseline characteristics

**Tables 1** and **2** list the baseline characteristics of the patients, which were well matched in the two groups. The mean CLQ score was 2.4 (SD 0.7, range 1–4) and 80% of the study population were women. The mean age was 53.1 (SD 12.8, range 21–88) and the mean body mass index (BMI) was 28.2 (SD 6.8, range 17.5–52); 62% of the patients had a BMI greater than 25, and in 34% the BMI was greater than 30. Furthermore, 56% of the patients had prior MR examinations which were prevented, aborted or performed with conscious sedation due to claustrophobia. According to the American College of Radiology guidelines [33], [34], [35], [36], [37], the indications for MR imaging were not inappropriate in any of the patients (**Table 1**).

**Table 2:** Characteristics of the 174 patients enrolled in the study.

	Short-bore MR group (n = 87)	Open MR group (n = 87)	*P* Value
**Characteristics**			
Female sex	70 (81)	69 (79)	0.85
Age	54.3 (SD 12.7)	52 (SD 12.9)	0.23
BMI*	27.5 (SD 6.8)	29 (SD 6.7)	0.15
Body circumferences in cm†			
Maximum circumference	111.3 (SD 15.6)	114.9 (SD 14)	0.12
Chest circumference	104.2 (SD 16.3)	107.5 (SD 14.9)	0.17
Waist circumference	95.9 (SD 19.8)	100.3 (SD 17.8)	0.13
Hip circumference	110 (SD 15)	113.8 (SD 13.4)	0.09
Medication‡			
Pain medication	34 (39)	38 (44)	0.54
Antidepressant medication	12 (14)	15 (17)	0.53
Sedative medication	7 (8)	7 (8)	1
Outpatients	79 (91)	83 (95)	0.23
EQ VAS§	57.1 (SD 20.1)	53.7 (SD 19.3)	0.27
**Socioeconomic characteristics**			
Unemployed	12 (14)	17 (20)	0.31
Pensioner	28 (32)	25 (29)	0.62
Years in school	10.1 (SD 1.7)	10.3 (SD 1.6)	0.49
State health insurance	82 (94)	82 (94)	1
Private health insurance	5 (6)	5 (6)	1

Values are arithmetic mean (standard deviation [SD]) or number (%). Percentages may not total 100% because of rounding.

*The body mass index (BMI) is the weight in kilograms divided by the square of the height in meters.

† Body circumferences were available for 80 patients in the short-bore MR group and 85 patients in the open MR group.

‡ Pain medication comprises regular use of cyclo-oxigenase inhibitors, acetaminophen, or opioids. Antidepressant medication comprises regular use of tricyclic antidepressants, selective serotonin reuptake inhibitors, or opipramol. Sedative medication comprises regular use of benzodiazepines or other sedatives.

§ The EuroQol (EQ-5D) is a self-report questionnaire for assessment of general health-related quality of life [30]. It includes a vertical visual analogue scale (EQ VAS) ranging from 0 (worst imaginable) to 100 (best imaginable) to rate the current state of health.

### Main outcome measures

Of the 174 enrolled patients, two had to be withdrawn from the study before imaging due to first-trimester pregnancy (relative contraindication) and urgent hospitalization (**Figure 1**). Thus, 172 patients were included in the intention-to-treat analysis. There were 33 claustrophobic events in the short-bore MR group (n = 85, 39%, [95% CI 28% to 50%]), compared to 23 claustrophobic events in the open MR group (n = 87, 26% [95% CI 18% to 37%]; absolute risk reduction of 12% and relative risk reduction of 32%; *P* = 0.08). This difference also remained non-significant in subgroup analyses (patient characteristics and the anatomical region being examined). Among the 53 patients who could not complete imaging in the per-protocol analysis and who were cross-referred to the other scanner (**Figure 1**), there were 19 claustrophobic events for short-bore (n = 23, 83% [95% CI 61% to 95%]) and 21 claustrophobic events for open MR (n = 33, 70% [95% CI 51% to 85%]; *P* = 0.35). Patients who did not tolerate the second MR scanner either were sedated using a mean of 2.6 mg (SD 1) midazolam IV (sedation success rate of 100% [26 of 26]; no adverse events, **Figure 1**) [25].

### Duration of MR imaging and course of events

Patients who completed their MR examination were in the examination room for 31.7 min (SD 13.2) in the short-bore group and for 42.1 min (SD 19.5) in the open group (*P* = 0.001). Patients who had a claustrophobic event remained in the examination room for 3.8 min (SD 4.4) in the short-bore group and for 8.5 min (SD 7) in the open group (*P* = 0.004). This difference was due to a significantly larger number of patients who already rejected imaging when entering the examination room in the short-bore group (33% versus 4%; *P* = 0.01; **Table 3**). In the open MR group there was only one event when entering the examination room (4% [95% CI 0% to 22%]), whereas there were 22 during positioning and imaging (96% [95% CI 78% to 100%]; **Table 3**). Analysis of the total time required to successfully complete the MR examination (one or two sessions) also revealed a significantly shorter time for the short-bore group (33.4 min [SD 18.9] versus 39.1 min [SD 20]; *P* = 0.03).

**Table 3:** Occurrence of claustrophobic events during different phases of the MR procedure.

	Intention-to-treat analysis			Per-protocol analysis		
	Short-bore MR group	Open MR group	*P* Value	Short-bore MR group	Open MR group	*P* Value
	n/total n (% [95% CI])			n/total n (% [95% CI])		
**First MR imaging**						
Total*	33/85 (39 [28 to 50])	23/87 (26 [18 to 37])	0.08	30/82 (37 [26 to 48])	23/87 (26 [18 to 37])	0.16
Entering the examination room	11/33 (33 [18 to 51])	1/23 (4 [0 to 22])	0.01	11/30 (37 [20 to 56])	1/23 (4 [0 to 22])	0.007
During positioning on the scanner table†	17/33 (52 [34 to 69])	20/23 (87 [66 to 97])	0.009	17/30 (57 [37 to 75])	20/23 (87 [66 to 97])	0.03
During MR imaging	2/33 (6 [1 to 20])	2/23 (9 [1 to 28])	1	2/30 (7 [1 to 22])	2/23 (9 [1 to 28])	1
**MR imaging after cross-referral**						
Total*	19/23 (83 [61 to 95])	21/30 (70 [51 to 85])	0.35	12/16 (75 [48 to 93])	18/27 (67 [46 to 84])	0.73
Entering the examination room	7/19 (37 [16 to 62])	3/21 (14 [3 to 36])	0.15	7/12 (58 [28 to 85])	3/18 (17 [4 to 41])	0.045
During positioning on the scanner table†	4/19 (21 [6 to 46])	15/21 (71 [48 to 89])	0.002	4/12 (33 [10 to 65])	15/18 (83 [59 to 96])	0.009
During MR imaging	1/19 (5 [0 to 26])	0/21 (0 [0 to 16])	0.47	1/12 (8 [0 to 39])	0/18 (0 [0 to 19])	0.4

Values are numbers (%) and 95% confidence intervals (CI). Percentages may not total 100% because of rounding. P values were obtained using the chi-squared and Fisher's exact test. See **Figure 1** for the flow of patients.

*Patients who did not attend their MR appointment due to claustrophobia were classified as having an event for the intention-to-treat analysis but were not otherwise categorized.

† Including preparation of the patients on the MR table.

### Prediction of events by psychological instruments and patient characteristics

Patients with a claustrophobic event had a total mean CLQ score of 2.6 (SD 0.7), compared to 2.3 (SD 0.7) for patients without an event (*P* = 0.009). This was mainly due to a relevant difference on the CLQ suffocation subscale (2.3 [SD 0.9] versus 1.8 [SD 0.8]; *P* = 0.003). None of the other questionnaire scores [26], [27], [28], [29], [30] were significantly associated with events. Subgroup analyses showed significantly more events in patients with prior prevented or aborted MR (**Table 4**). However, there were no differences of the total mean CLQ scores between patients with and without prior prevented or aborted MR (2.4 [SD 0.7] versus 2.3 [0.7]; *P* = 0.4). Furthermore, patients with an event had a mean visual analogue scale score of 70.9 (SD 19.9), compared to 61.7 (SD 25.6) for patients without an event (*P* = 0.03). There were no differences between men and women (**Table 4**).

**Table 4:** Event rates in subgroup analyses.

	Intention-to-treat analysis			Per-protocol analysis		
	No event	Event	*P* Value	No event	Event	*P* Value
**First MR imaging**						
Age	52 (SD 13)	56 (SD 13)	0.1	52 (SD 13)	55 (SD 13)	0.16
BMI*	28 (SD 7)	29 (SD 7)	0.19	28 (SD 7)	29 (SD 7)	0.18
Female sex	95/116 (82 [74 to 88])	42/56 (75 [62 to 86])	0.29	95/116 (82 [74 to 88])	40/53 (76 [62 to 86])	0.33
Prior prevented or aborted MRI†	57/116 (49 [40 to 59])	40/56 (71 [58 to 83])	0.006	57/116 (49 [40 to 59])	39/53 (74 [60 to 85])	0.003
Feet-first MRI‡	40/116 (35 [26 to 44])	20/56 (36 [23 to 50])	0.87	40/116 (35 [26 to 44])	18/53 (34 [22 to 48])	0.95

Values are arithmetic mean (standard deviation [SD]) or number (%) and 95% confidence intervals (CI). Percentages may not total 100% because of rounding. P values were obtained using the chi-squared and Fisher's exact test. See **Figure 1** for the flow of patients. Patients who did not attend their MR imaging appointment were classified as having an event for the intention-to-treat analysis but not for the per-protocol analysis.

*The body mass index (BMI) is the weight in kilograms divided by the square of the height in meters.

† Including MR imaging which was prevented, aborted or performed with conscious sedation due to claustrophobia.

‡ For MR imaging of the cervical spine, the shoulder, and the head, patients were examined head-first because feet-first imaging, which alleviates claustrophobia by a factor of more than 10, was possible only for lumbar spine imaging in both groups [12].

### Pre-imaging anxiety

Directly before MR imaging, the State questionnaire of the Spielberger State-Trait Anxiety Inventory (STAI) was used to assess patients' state anxiety [27]. Among the 43 patients who had two MR appointments (**Figure 1**), the state anxiety before MR imaging did not change significantly from the first to the second appointment (2.7 [SD 0.6] versus 2.8 [SD 0.6]; *P* = 0.2). Subgroup analyses assessing patients with an event and patients with prior prevented or aborted MR examinations did also not show relevant differences in state anxiety in comparison with the other patients (**Table 5**), as well as in comparison of the two appointments.

**Table 5:** Pre-imaging state anxiety assessed using the State questionnaire of the STAI [27] mean score in patients who also had second MR imaging after cross-referral.

	First MR imaging		*P* Value	Second MR imaging		*P* Value
	Yes (n = 30)	No (n = 13)		Yes (n = 30)	No (n = 13)	
Event	2.6 (SD 0.9)	2.6 (SD 0.7)	1	2.9 (SD 0.6)	2.6 (SD 0.5)	0.2
Prior prevented or aborted MRI*	2.6 (SD 0.7)	2,6 (SD 0.8)	0.45	2.9 (SD 0.6)	2.7 (SD 0.7)	0.4

Values are arithmetic mean (standard deviation [SD]). P values were obtained using the unpaired t-test. Of the 43 patients who had a second MR appointment, 30 had an event and also 30 patients had prior prevented or aborted MR examinations. Thirteen of the 56 patients with an event did not attend the second MR imaging appointment because of claustrophobia. See **Figure 1** for the flow of patients.

*Including MR imaging which was prevented, aborted or performed with conscious sedation due to claustrophobia.

### Clinical follow-up

The response-rate for clinical follow-up after seven months was 78% (134/172). The MR examinations yielded new findings that explained symptoms at presentation in 69% of cases and confirmed known findings in 17% of cases; no MR was non-diagnostic. 47% of MR examinations led to changes in medical treatment, and in 10% surgery was initiated. At follow-up, 47% reported an improvement of their medical condition, in 48% the condition was unchanged, and 7 patients had a deterioration of their health status (e.g., diagnosis of Alzheimer's disease, recurrent low back pain after pregnancy).

### Psychological follow-up

At follow-up, the CLQ was completed by 73% (125/172) of the study participants. The mean overall CLQ score at follow-up was significantly lower (2.2 [SD 0.7]) compared with initial assessment (initial mean CLQ score of patients who responded at follow-up: 2.3 [SD 0.79]; *P* = 0.018). This was mainly due to a significant reduction of the CLQ suffocation subscale score (n = 125, 1.7 [SD 0.9] versus 1.9 [SD 0.8]; *P* = 0.003). However, in patients with events the mean overall CLQ score at follow-up was not significantly different compared with initial assessment (2.4 [SD 0.8] versus 2.5 [SD 0.7]; *P* = 0.55). Furthermore, patients had significantly reduced mean scores on the claustrophobia visual analogue scale assessing perceived claustrophobia at seven months (56.9 [SD 24.3] versus 62.8 [SD 24.5]; *P* = 0.003). But, in patients with events, perceived claustrophobia increased at follow-up in 32%, while only 11% of patients without events reported such an increase (*P* = 0.004).

Patients were also asked at follow-up to rate their pre-imaging anxiety using the State questionnaire in retrospect [27]. For the first MR appointment, these data were available for 116 patients (29 of them with an event). These 116 patients had a significantly higher mean score in the State questionnaire addressing the first MR appointment at follow-up compared with the assessment directly before MR imaging (3 [SD 1.6] compared to 2.6 [SD 0.7]; *P* = 0.003). For the second appointment, there were no significant differences in the available data of 28 patients.

## Discussion

### Principal findings and interpretation

Recent short-bore and open panoramic scanners have the potential to reduce claustrophobia which is a common problem in MR imaging [11], [12], [19], [20], [38]. In this first randomized controlled trial on claustrophobia in MRI both, short-bore and open, scanners showed disappointing event rates of more than 25%, irrespective of patient characteristics and the anatomical region being examined. Certainly, the surprisingly high event rate for both scanners is at least partly due to our rather high-risk patient population that had a CLQ mean score of 2.4 (SD 0.7) which is comparable to other high-risk groups, e.g. claustrophobic students (CLQ of 2.0 [SD 0.6]) [23]. About 80% of the study population were women who have been shown to be more likely to experience claustrophobia during MR imaging (hazard ratio of 1.8) [3], [5], [12], [39], [40]. Moreover, over 80% of our patients had prior MR imaging experience and 98 patients (56%) already had claustrophobic events leading to prevention, abortion, or requiring sedation for completion of prior MR (90 conventional long narrow-bore, 6 short-bore, 2 open panoramic systems). Previous unpleasant MR experiences have been shown to be associated with higher pre-imaging anxiety and thus higher event rates [39], [41], [42] which were also found in the 56% of our patients who had prior prevented or aborted MR: 71% of patients with events had prior negative MR experiences, compared to 49% of patients without events (**Table 4**). Still, the pre-imaging anxiety on the State questionnaire of the STAI [27] was not higher in patients with prior negative MR experiences (**Table 5**).

Although the event rates indicate a potential benefit of open scanners, these examinations, weather or not completed, took significantly longer. In patients who could not complete the examination, this was due to the fact that the claustrophobic events occurred earlier in the short-bore group as there were significantly more patients who had events already when entering the examination room. From a practical perspective, it may be a considerable advantage to detect events earlier. However, in both groups the majority of patients with events (short-bore group 52%, open MR group 87%) refused to undergo MR imaging during positioning on the MR table. Most of these patients reported severe panic while the table was moved into the MR scanner so that the final position could not be reached. Others reached the final position but could not tolerate it long enough. Some patients already reported severe panic during positioning of the MR surface coils and refused to continue. This highlights that the most problematic phase of the scan procedure is during positioning, as well as on entry in the examination room. Thus, procedural modifications might be influential for reduction of claustrophobic events. In our study, the MR imaging procedure was kept constant and all patients were told that positioning of the table could be repeated (thus moving the table in and out of the MR scanner) so that they could get accustomed to the situation. The significantly longer imaging duration in the open MR group was mainly attributable to longer sequence acquisition times [21] due to different field strengths and gradients.

Concerning the prediction of claustrophobic events by psychological instruments, in our study, the suffocation subscale of the CLQ was found to be the best discriminator. Thus, the CLQ might be a useful tool to identify patients at increased risk for claustrophobia during MR imaging which allows for early interventions such as by relaxation techniques [15], social support or conscious sedation.

At seven-months follow-up, 86% of all scanned patients reported that their clinical symptoms were explained by findings at MR imaging. Furthermore, 47% reported an improved medical condition at follow-up, which may at least in part be due to adequate MR referrals according to the American College of Radiology guidelines in all patients [33], [34], [35], [36], [37]. Thus, our results support previous findings which show that adherence to referral guidelines is pivotal considering MRI's limited diagnostic yield for instance in patients referred for lumbar spine radiographs [43] or without any back pain [44]. Furthermore, all completed MR examinations had diagnostic image quality.

Assessing claustrophobia after seven months, patients had reduced mean scores on the claustrophobia visual analogue scale and the CLQ, which is consistent with reports of decreased anxiety after completed MR examinations [42], [45] and highlights the potential of exposure therapy to reduce claustrophobia [14], [15], [46]. However, anxiety during MR imaging can also increase or even induce claustrophobia after the examination [39], [42], [47], [48], [49], [50], which was reported by 32% of our patients with events. Interestingly, patients rated their pre-imaging anxiety at the first MR appointment significantly higher in retrospect at seven-month follow-up compared with the assessment directly before MR imaging.

### Strengths and limitations

This is the first trial directly comparing short-bore and open MR imaging with regard to reduction of claustrophobia as well as diagnostic utility. Strengths of our study include the random assignment of patients to one of the two scanners and the inclusion of psychological instruments. We decided to include only patients with an increased risk to suffer from claustrophobia in MR imaging, because these patients should be addressed when more patient-centered MR scanners are developed. Furthermore, for the power analysis we used published non-randomized studies which suggested an advantage of open MR imaging [19], [20].

Our study has also limitations. It is a single-center study with two MR scanners in a specific environment, which may affect its generalizability. However, we believe that our results are likely to be generalizable to other MR scanners with a similar design approach. Furthermore, neither patients nor assessors could be blinded to the study group because of the MR imaging setting. Further potential limitations require discussion. First, our results did not show the superiority of open MR imaging that this study was powered to detect based on data from recent non-randomized trials [12], [19], [20]. Recalculating the power of our study we note that with a 33% average event rate, true differences of 20% (23% versus 43%) achieve sufficient power for 174 patients. Observed differences of 14% (26% versus 40%) would have been significant. Second, the study design with the option of undergoing a second MR examination after cross-referral can be discussed as one reason for the high number of claustrophobic events in both study groups (26 and 39%). However, this also meant a second appointment for imaging with additional efforts for the patients. Third, our negative results may serendipitously point to more salient factors to explain why so many scans were prevented or aborted. Undoubtedly, there are several factors which can contribute to anxiety during MR imaging such as pain, noise, previous unpleasant MR experiences, concern about possible diagnostic findings, the examination duration, and symptoms of depression [2], [3], [4], [39], [41], [42], [51], [52], while feet-first positioning can alleviate claustrophobia by a factor of more than 10 [12]. These and other influencing factors were assessed or tempted to be kept constant in our study. In subgroup analyses, there were no relevant differences comparing the two groups and in patients with and without events, except for assessing subjective pain levels and the examination duration. Patients reported significantly (*P* = 0.01) higher pain levels during the examinations in the open MR group (**Table 1**). However, it is unlikely that these factors had a relevant influence on the event rates as pain usually occurs in the course of the MR examination and there were only two patients in each study group who had a claustrophobic event during and not before imaging. We also found that patients with prior negative MR experiences had significantly more claustrophobic events. Other factors have not shown significant influence on event rates (**Table 4**). Fourth, several studies have shown the importance of support by nursing staff and technicians [20], [51], [53], [54]. It might have influenced patients that they knew that the staff was aware of their anxiety. In order to keep the influence as constant as possible, two nurses supported the study. Furthermore, the technicians were instructed to support the patients as in clinical routine avoiding being overly protective to reflect clinical reality. Last, it should be mentioned that there are now MR scanners with a slightly shorter (1.25 m) and wider bore (0.7 m; Siemens Magnetom Espree) available [55].

### Comparison with other studies

Several non-randomized studies have shown the potential of recent high-field short-bore and open panoramic MR scanners to reduce claustrophobia. A recent study by Bangard et al. concluded that open MR imaging has great potential for reducing claustrophobic events [19]. In 36 claustrophobic patients, the scan termination rate was reduced to 8% compared to 56% in previous conventional closed-bore imaging in the same patients. In a study by Spouse et al., 96% of 50 claustrophobic patients, who were unable to complete a conventional closed-bore MR scan, successfully underwent imaging on an open interventional MR scanner with a gap in the bore of the magnet (“double doughnut”) [20]. However, friends or relatives were allowed to stay in the magnet room and many patients indicated that this, beside the scanner design, had helped them considerably. Other clinical studies have investigated the potential of short-bore MR scanners to reduce claustrophobia. Dewey et al. compared a short- with a closed-bore scanner in 55,734 consecutive patients and found the short-bore scanner to reduce claustrophobic events by a factor of 3 [12]. In contrast, Dantendorfer et al. found no significant difference in the occurrence of claustrophobic events in a retrospective study on 5,682 patients examined in either a short- or a closed-bore MR scanner [11]. However, they discussed a selection bias because staff was referring highly anxious patients for examination on the short-bore scanner. Compared to our study, the reported trials were not randomized and not comparing different MR scanners with more patient-centered designs. No study assessed at which point in the MR imaging procedure claustrophobia did occur. Moreover, some of the results are difficult to interpret because of methodological weaknesses such as selection bias.

Regarding the predictive value of the CLQ, in a study by McIsaac et al. in 80 MR-naïve outpatients, CLQ scores significantly discriminated between patients who experienced claustrophobia during MR imaging and those who did not [50]. McGlynn et al. showed CLQ suffocation subscale scores to strongly predict self-reported subjective fear in 64 students who were exposed to a mock MR procedure [56]. Our results support these findings and our study was the first correlating CLQ scores with claustrophobic events precluding MR imaging rather than with subjective fear.

### Future research

Based on our findings, future clinical research should investigate more patient-centered MR scanner designs and their potential to further alleviate claustrophobia. A promising approach is the development of open mobile MR sensors [57], [58]. However, low and inhomogeneous magnetic fields still pose challenges for clinical applicability of such systems. Our results highlight that the most problematic phase of the scan procedure is during positioning, as well as on entry into the examination room. Thus, procedural modifications might also be influential for reduction of claustrophobic event rates. To better elucidate the predictive value of the CLQ for identification of patients who will have events, it should be investigated in larger populations of unselected patients.

### Conclusions

In conclusion, the present study in high-risk patients demonstrated claustrophobia precluding MR imaging in more than 25% of examinations despite using scanner designs expected to lower the rate of claustrophobic events. Although the results support an advantage of open MR, events did occur earlier in the imaging procedure in the short-bore group, which can facilitate interventions and prevent waste of valuable examination time. The CLQ may be a useful tool to detect patients at risk before claustrophobia occurs. The majority of patients who are affected are women (80% of our study population). Further developments towards a more patient-centered MR scanner environment are clearly needed to make this important diagnostic test available to all patients.

## Supporting Information

Checklist S1 CONSORT checklist.
(DOC)

## References

[1] Fuchs VR, Sox HC (2001). Physicians' views of the relative importance of thirty medical innovations.. Health Aff (Millwood).

[2] Melendez JC, McCrank E (1993). Anxiety-related reactions associated with magnetic resonance imaging examinations.. Jama.

[3] Katz RC, Wilson L, Frazer N (1994). Anxiety and its determinants in patients undergoing magnetic resonance imaging.. J Behav Ther Exp Psychiatry.

[4] Quirk ME, Letendre AJ, Ciottone RA, Lingley JF (1989). Anxiety in patients undergoing MR imaging.. Radiology.

[5] Berg WA, Blume JD, Adams AM, Jong RA, Barr RG (2010). Reasons women at elevated risk of breast cancer refuse breast MR imaging screening: ACRIN 6666.. Radiology.

[6] Brennan SC, Redd WH, Jacobsen PB, Schorr O, Heelan RT (1988). Anxiety and panic during magnetic resonance scans.. Lancet.

[7] Kortepeter MG (1991). MRI: my resonant image.. Ann Intern Med.

[8] Bluemke DA, Breiter SN (2000). Sedation procedures in MR imaging: safety, effectiveness, and nursing effect on examinations.. Radiology.

[9] Malviya S, Voepel-Lewis T, Eldevik OP, Rockwell DT, Wong JH (2000). Sedation and general anaesthesia in children undergoing MRI and CT: adverse events and outcomes.. Br J Anaesth.

[10] Berlin L (2001). Sedation and analgesia in MR imaging.. AJR Am J Roentgenol.

[11] Dantendorfer K, Wimberger D, Katschnig H, Imhoff H (1991). Claustrophobia in MRI scanners.. Lancet.

[12] Dewey M, Schink T, Dewey CF (2007). Claustrophobia during magnetic resonance imaging: cohort study in over 55,000 patients.. J Magn Reson Imaging.

[13] Sarji SA, Abdullah BJ, Kumar G, Tan AH, Narayanan P (1998). Failed magnetic resonance imaging examinations due to claustrophobia.. Australas Radiol.

[14] Booth R, Rachman S (1992). The reduction of claustrophobia–I.. Behav Res Ther.

[15] Quirk ME, Letendre AJ, Ciottone RA, Lingley JF (1989). Evaluation of three psychologic interventions to reduce anxiety during MR imaging.. Radiology.

[16] Lang EV, Ward C, Laser E (2010). Effect of team training on patients' ability to complete MRI examinations.. Acad Radiol.

[17] Lang EV, Benotsch EG, Fick LJ, Lutgendorf S, Berbaum ML (2000). Adjunctive non-pharmacological analgesia for invasive medical procedures: a randomised trial.. Lancet.

[18] Lang EV, Berbaum KS, Faintuch S, Hatsiopoulou O, Halsey N (2006). Adjunctive self-hypnotic relaxation for outpatient medical procedures: a prospective randomized trial with women undergoing large core breast biopsy.. Pain.

[19] Bangard C, Paszek J, Berg F, Eyl G, Kessler J (2007). MR imaging of claustrophobic patients in an open 1.0T scanner: motion artifacts and patient acceptability compared with closed bore magnets.. Eur J Radiol.

[20] Spouse E, Gedroyc WM (2000). MRI of the claustrophobic patient: interventionally configured magnets.. Br J Radiol.

[21] Enders J, Zimmermann E, Rief M, Martus P, Klingebiel R (2011). Reduction of claustrophobia during magnetic resonance imaging: methods and design of the “CLAUSTRO” randomized controlled trial.. BMC Med Imaging.

[22] Moher D, Jones A, Lepage L (2001). Use of the CONSORT statement and quality of reports of randomized trials: a comparative before-and-after evaluation.. Jama.

[23] Radomsky AS, Rachman S, Thordarson DS, McIsaac HK, Teachman BA (2001). The Claustrophobia Questionnaire.. J Anxiety Disord.

[24] Dewey M, Schink T, Dewey CF (2007). Frequency of referral of patients with safety-related contraindications to magnetic resonance imaging.. Eur J Radiol.

[25] No Authors listed (1996). Practice guidelines for sedation and analgesia by non-anesthesiologists. A report by the American Society of Anesthesiologists Task Force on Sedation and Analgesia by Non-Anesthesiologists.. Anesthesiology.

[26] Chambless DL, Caputo GC, Bright P, Gallagher R (1984). Assessment of fear in agoraphobics: the body sensations questionnaire and the agoraphobic cognitions questionnaire.. J Consult Clin Psychol.

[27] Spielberger CD (1983). Manual for the State-Trait Anxiety Inventory (STAI).

[28] Wolpe J, Lang PJ (1977). Manual for the Fear Survey Schedule (rev ed.).

[29] Beck AT, Steer RA, Brown GK (1996). Manual for Beck Depression Inventory II (BDI-II).

[30] No Authors listed (1990). EuroQol-a new facility for the measurement of health-related quality of life. The EuroQol Group.. Health Policy.

[31] Altman D, Machin D, Bryant T, Gardner M (2000). Statistics with Confidence.

[32] Altman D, Machin D, Bryant T, Gardner M (2000). Statistics with Confidence.

[33] ACR Appropriateness Criteria® Acute Shoulder Pain. American College of Radiology Web Site.. http://www.acr.org/SecondaryMainMenuCategories/quality_safety/app_criteria/pdf/ExpertPanelonMusculoskeletalImaging/AcuteShoulderPain.aspx.

[34] ACR Appropriateness Criteria® Chronic Neck Pain. American College of Radiology Web Site.. http://www.acr.org/SecondaryMainMenuCategories/quality_safety/app_criteria/pdf/ExpertPanelonMusculoskeletalImaging/ChronicNeckPainDoc9.aspx.

[35] ACR Appropriateness Criteria® Low Back Pain. American College of Radiology Web site.. http://www.acr.org/SecondaryMainMenuCategories/quality_safety/app_criteria/pdf/ExpertPanelonNeurologicImaging/LowBackPainDoc7.aspx.

[36] ACR Appropriateness Criteria® Headache. American College of Radiology Web site.. http://www.acr.org/SecondaryMainMenuCategories/quality_safety/app_criteria/pdf/ExpertPanelonNeurologicImaging/HeadacheDoc6.aspx.

[37] ACR Appropriateness Criteria® Vertigo and Hearing Loss. American College of Radiology Web site.. http://www.acr.org/SecondaryMainMenuCategories/quality_safety/app_criteria/pdf/ExpertPanelonNeurologicImaging/VertigoandHearingLossDoc14.aspx.

[38] Dantendorfer K, Amering M, Bankier A, Helbich T, Prayer D (1997). A study of the effects of patient anxiety, perceptions and equipment on motion artifacts in magnetic resonance imaging.. Magn Reson Imaging.

[39] Lukins R, Davan IG, Drummond PD (1997). A cognitive behavioural approach to preventing anxiety during magnetic resonance imaging.. J Behav Ther Exp Psychiatry.

[40] Dewey M (2010). Claustrophobia preventing MR imaging of the breast.. Radiology.

[41] MacKenzie R, Sims C, Owens RG, Dixon AK (1995). Patients' perceptions of magnetic resonance imaging.. Clin Radiol.

[42] Kilborn LC, Labbé EE (1990). Magnetic resonance imaging scanning procedures: development of phobic response during scan and at one-month follow-up.. J Behav Med.

[43] Jarvik JG, Hollingworth W, Martin B, Emerson SS, Gray DT (2003). Rapid magnetic resonance imaging vs radiographs for patients with low back pain: a randomized controlled trial.. Jama.

[44] Jensen MC, Brant-Zawadzki MN, Obuchowski N, Modic MT, Malkasian D (1994). Magnetic resonance imaging of the lumbar spine in people without back pain.. N Engl J Med.

[45] Harris LM, Robinson J, Menzies RG (1999). Evidence for fear of restriction and fear of suffocation as components of claustrophobia.. Behav Res Ther.

[46] Marks I (1991). Phobias and related anxiety disorder.. Bmj.

[47] Fishbain D, Goldberg H, Labbé E, Zacher D, Steele-Rosomoff R (1989). MR imaging as a trigger for persistent claustrophobia.. AJR Am J Roentgenol.

[48] Fishbain DA, Goldberg M, Labbe E, Zacher D, Steele-Rosomoff R (1988). Long-term claustrophobia following magnetic resonance imaging.. Am J Psychiatry.

[49] Kanal E, Shellock FG, Talagala L (1990). Safety considerations in MR imaging.. Radiology.

[50] McIsaac HK, Thordarson DS, Shafran R, Rachman S, Poole G (1998). Claustrophobia and the magnetic resonance imaging procedure.. J Behav Med.

[51] Granet RB, Gelber LJ (1990). Claustrophobia during MR imaging.. N J Med.

[52] Vitaliano PP, DeWolfe DJ, Maiuro RD, Russo J, Katon W (1990). Appraised Changeability of a Stressor as a Modifier of the Relationship Between Coping and Depression: A Test of the Hypothesis of Fit.. J Pers Soc Psychol.

[53] Törnqvist E, Mansson A, Larsson EM, Hallström I (2006). It's like being in another world - Patients' lived experience of magnetic resonance imaging.. J Clin Nurs.

[54] Carter MM, Hollon SD, Carson R, Shelton RC (1995). Effects of a safe person on induced distress following a biological challenge in panic disorder with agoraphobia.. J Abnorm Psychol.

[55] Adamietz B, Cavallaro A, Radkow T, Alibek S, Holter W (2007). [Tolerance of magnetic resonance imaging in children and adolescents performed in a 1.5 Tesla MR scanner with an open design].. Rofo.

[56] McGlynn FD, Smitherman TA, Hammel JC, Lazarte AA (2007). Component fears of claustrophobia associated with mock magnetic resonance imaging.. J Anxiety Disord.

[57] Meriles CA, Sakellariou D, Heise H, Moule AJ, Pines A (2001). Approach to high-resolution ex situ NMR spectroscopy.. Science.

[58] Blumich B, Blumler P, Eidmann G, Guthausen A, Haken R (1998). The NMR-mouse: construction, excitation, and applications.. Magn Reson Imaging.

